# Understanding Magnetic Resonance Imaging in Multiple Sclerosis (UMIMS): Development and Piloting of an Online Education Program About Magnetic Resonance Imaging for People With Multiple Sclerosis

**DOI:** 10.3389/fneur.2022.856240

**Published:** 2022-03-28

**Authors:** Magalie Freund, Insa Schiffmann, Anne Christin Rahn, Declan Chard, Carsten Lukas, Jutta Scheiderbauer, Anna Sippel, Christoph Heesen

**Affiliations:** ^1^Department of Neurology, Institute of Neuroimmunology and Multiple Sclerosis, University Medical Centre Hamburg-Eppendorf, Hamburg, Germany; ^2^Department of Neurology, University Medical Centre Hamburg-Eppendorf (UKE), Hamburg, Germany; ^3^Institute for Social Medicine and Epidemiology, Nursing Research Unit, University of Lübeck, Lübeck, Germany; ^4^NMR Research Unit, Queen Square MS Centre, Department of Neuroinflammation, UCL Institute of Neurology, Faculty of Brain Sciences, University College London, London, United Kingdom; ^5^National Institute for Health Research (NIHR), University College London Hospitals (UCLH), Biomedical Research Centre, London, United Kingdom; ^6^Department of Diagnostic and Interventional Radiology and Nuclear Medicine, Institute of Neuroradiology, St. Josef Hospital, Ruhr-University Bochum, Bochum, Germany; ^7^Department of Diagnostic and Interventional Radiology and Nuclear Medicine, St. Josef Hospital, Ruhr-University Bochum, Bochum, Germany; ^8^Foundation for Self-Determination and Self-Representation for People With MS, Trier, Germany

**Keywords:** magnetic resonance imaging, MRI-risk knowledge, multiple sclerosis, online education, pwMS, shared decision making

## Abstract

**Background:**

People with multiple sclerosis (pwMS) lack sufficient magnetic resonance imaging (MRI) knowledge to truly participate in frequently occurring MRI-related therapy decisions. An evidence-based patient information (EBPI) about MRI is currently lacking.

**Objective:**

The aim of this study was to develop an evidence-based online education program about limitations and benefits of MRI for pwMS. Ultimately, our goal was to improve MRI risk-knowledge, empower pwMS, and promote shared decision-making.

**Methods:**

The program's contents were based on literature research and a previous pilot study. It was revised following 2 evaluation rounds with pwMS, MRI experts and expert patients. In a pilot study, *n* = 92 pwMS received access to the program for 4 weeks. User experiences and acceptance, MRI knowledge (MRI-RIKNO 2.0 questionnaire) and emotions and attitudes toward MRI (MRI-EMA questionnaire) were assessed. Results were compared to a previous survey population of *n* = 508 pwMS without access to the program.

**Results:**

Participants rated the program as easy to understand, interesting, relevant, recommendable, and encouraging. In comparison to pwMS without access to the program, MRI risk-knowledge and perceived MRI competence were higher.

**Conclusion:**

Satisfaction with the program and good MRI-risk knowledge after usage demonstrates the need and applicability of EBPI about MRI in MS.

## Introduction

Magnetic resonance imaging (MRI) plays a crucial role in the diagnosis of multiple sclerosis (MS), (1) provides prognostic information (2) and is increasingly used as to monitor treatment efficacy and to determine treatment eligibility (3). However, beyond diagnosis, the specific role of MRI in clinical practice is controversial, (4, 5) and particular MRI results alone cannot currently be used to determine which of several medically viable therapeutic options is most likely to benefit a given person with MS (pwMS). In these cases, patients' personal perspectives become highly relevant.

Person with MS prefer an active role in the decision-making process (6). This requires sufficient disease-specific knowledge to enable them to fully participate in medical decision-making. However, pwMS' knowledge about their disease still has significant scope to improve: in a multi-national survey of *n* = 1,939 people with relapsing remitting MS an average of 41% of MS-specific knowledge questions were answered correctly; (7) MRI-specific knowledge in *n* = 508 pwMS was slightly better (62% correct answers) (8). In a pilot study using a face-to-face education program, participants demonstrated their learning potential by increasing their MRI-knowledge from 43 to 74% correctly answered questions in a MRI risk-knowledge questionnaire (9). PwMS also stated that they did not feel confident to discuss their MRI results with their physician, MRI is of high importance to them, and they were willing to spend several hours on MRI education (10). There is little information on MRI available for pwMS. However, pwMS regularly search the internet, especially when considering treatment initiation or change, but they often do not recognize reliable information (11).

The objective of this study was the development of an evidence-based online education program on MRI in MS and its evaluation in a pilot study. We hypothesize that using the education program will increase pwMS' MRI risk-knowledge and enable them to more actively participate in MRI-related medical decisions.

## Materials and Methods

### Development of the Online Education Program

Development of the online education program “Understanding MRI in MS” (UMIMS) was based on the non-systematic literature searches, work by Brand et al. (9) and 2 rounds of interviews with pwMS, MRI experts and expert patients (i.e., people affected by MS, who include experiential knowledge of the illness as well as pertinent awareness of diseases and academic involvement) (12).

The first draft of the website was modeled after a face-to-face educational program (9). Interviewees received access for 2 weeks and filled out a questionnaire with multiple-choice and free text questions concerning general appraisal of the website, relevance, time spent on it, understandability, and neutral presentation of the information. In semi-structured interviews using an interview guide, interviewees explained how they experienced the website. Interviews were audiotaped, transcribed, content was analyzed, and the website revised accordingly (see Table S1).

#### Structure of the Website

**Figure 1:**
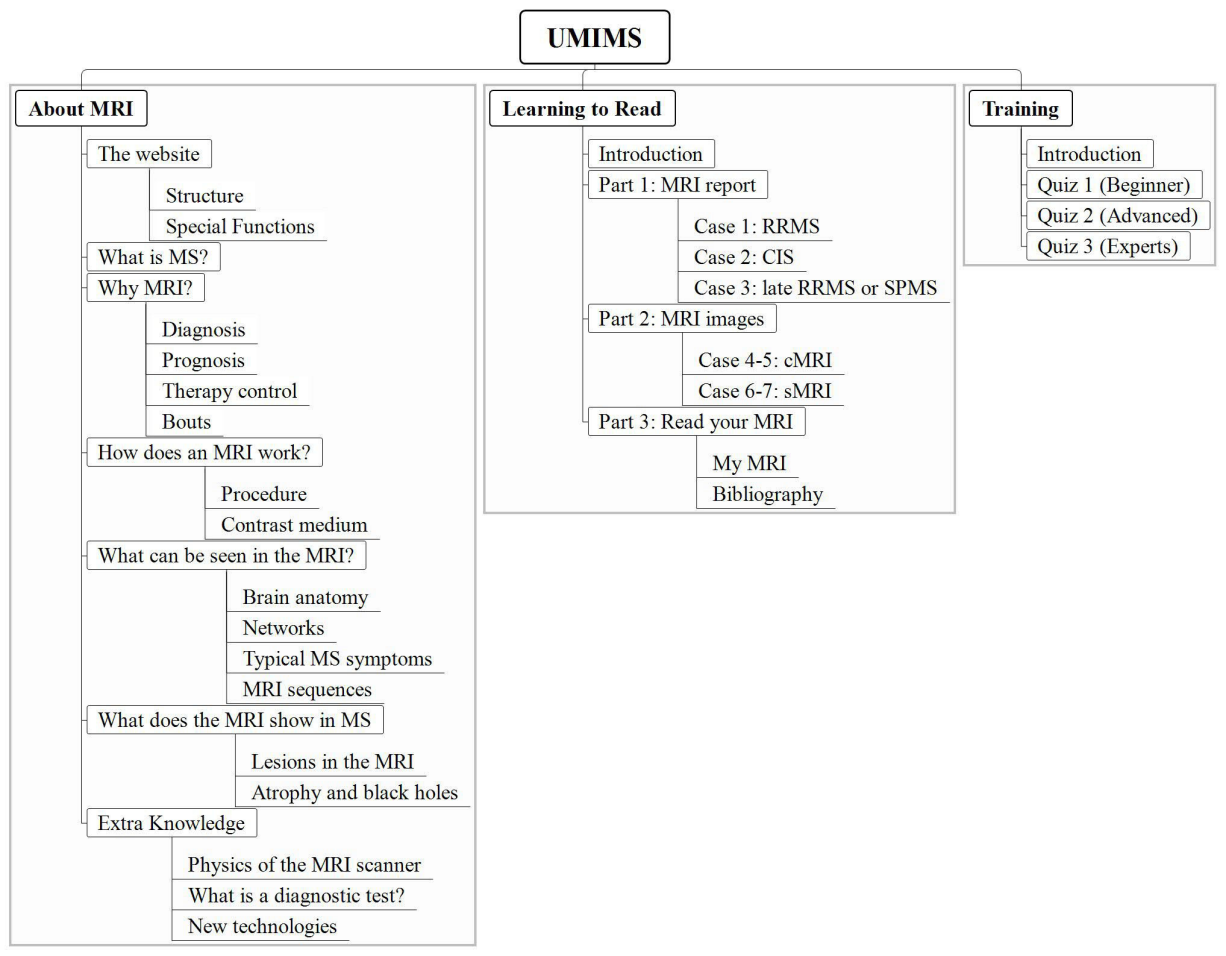
The structure of the online education program UMIMS. Users can click on the main headings “About MRI”, “Learning to read” and “Training” to open a drop-down menu with chapters (white boxes) and subchapters (underlined). UMIMS, Understanding MRI in MS.

Finally, UMIMS has 3 sections: “About MRI” covering educational chapters on MRI, “Learning to read” featuring an interactive MRI reading training with real MRI images and a quiz-section called “Training” (see Figure 1).

“About MRI” includes 6 chapters with 18 sub-chapters providing evidence-based information. Detailed information on the importance of MRI for diagnosis, prognosis, and therapy control in MS is given. In terms of limitations of MRI in MS pwMS get education on, e.g., sensitivity and specificity of diagnostic tests and the clinico-radiological paradox in MS.

Other chapters present with information on basic neuroanatomy, the MRI procedure, contrast agents, MRI sequences, lesion knowledge, and special knowledge (e.g., MRI physics and statistics). References are given with superscript numbers. Videos, easy-to-understand figures, and explanations of technical terms facilitate learning.

In “Learning to read” pwMS can train to understand their own MRI results using 3 different MRI reports translated into layman's terms and 7 original MRI images. The MRI images are accompanied by a step-by-step explanation for interpretation, questions on the shown information (e.g., how many lesions are visible) and corresponding answers. The sub-chapter “Read your MRI” encourages users to transfer their knowledge into real-life by giving instructions on using MRI viewers for one's own MRI images.

The “Training” Section consists of 3 quizzes, which are proven to enhance the learning process, (13) with increasing difficulty.

### Pilot Study

#### Study Design and Sampling

Understanding MRI in MS was piloted in a cross-sectional online survey with *n* = 92 pwMS. Participants were recruited from April 2018 until June 2018 *via* the newsletter of the MS day hospital of the University Medical Center Hamburg-Eppendorf (UKE) and the website of the German MS self-help organization. Participants were eligible, if they were over 18 years old and had clinically definite or suspected MS. Informed written consent was obtained online.

#### Measures and Data Collection

At baseline, participants provided demographic and disease-specific data (including Patient Determined Disease Steps [PDDS]) (14) and subjective MRI-knowledge (7-point Likert-scale ranging from 1 “I have no knowledge” to 7 “I have a lot of knowledge”). PwMS received access to UMIMS for 4 weeks and were invited to answer a follow-up questionnaire *via* e-mail reminders 2, 3, and 4 weeks after inclusion. Data collection was carried out using the survey platform Unipark (https://www.unipark.com/).

After using UMIMS, subjective MRI-knowledge as well as objective MRI risk-knowledge were assessed, the latter *via* the 14-item MRI risk-knowledge 2.0 (MRI-RIKNO 2.0) questionnaire (8) featuring questions concerning, e.g., basic neuroanatomy and relevance of MRI for prognosis (maximum score 22 points). Perception of the MRI procedure and results were assessed using the validated “EMotions and Attitudes toward MRI” (MRI-EMA)-questionnaire, (10) which consists of 10 items sorted into 4 subscales (Fear of MRI scan, Fear of MRI results, Feeling of control over the disease, Feeling of competence in the patient–physician encounter). Agreement to the items was assessed using 4-point Likert-scales ranging from strongly disagree (= 1) to strongly agree (= 4).

Acceptance, i.e., participants' experiences and interaction with UMIMS, was assessed *via* 11 items. For the first 5 items 7-point Likert-scales were used rating the program as comprehensible, new, relevant, encouraging, and creating curiosity. Scale values for, e.g., “comprehensible” ranged from 1 = *I did not understand the information on UMIMS at all* to 7 = *I understood the information on UMIMS completely*. For the next 5 items, participants evaluated whether the website was user-friendly, overwhelming, helpful, recommendable, and exhaustive on 4-point Likert-scales. The 4-point format was chosen to force respondents to take sides, ranging from strongly disagree (= 1) to strongly agree (= 4). The last item referred to the time spent on UMIMS, for which participants chose one option out of 7 (“no time at all” to “>8 h”).

Finally, participants were invited to give feedback *via* free-text comments. Comments were categorized by a research assistant into 3 categories: appraisal, criticism, and neutral comments. The website was revised accordingly.

#### Statistical Analysis

Data were analyzed descriptively using SPSS Statistics (version 23). Missing values were imputed for the MRI-EMA (*n* = 6 cases) and acceptance questionnaire (*n* = 8 cases); for the MRI-RIKNO 2.0 missing values were counted as incorrect answers.

In this paper, results of the MRI-RIKNO 2.0 and MRI-EMA of this cohort (*n* = 92 pwMS, referred to as “pilot cohort”) are contrasted to the results of a previous survey using the same questionnaires among a cohort of *n* = 508 pwMS without MRI education (“online cohort”) (8). Pearson-correlation analyses were performed to uncover whether (1) subjective knowledge, (2) time spent on the website, and (3) subjective degree of comprehension correlated with objective MRI risk-knowledge (i.e., results of the MRI-RIKNO 2.0). A correlation was considered small for *r* = 0.1, medium for *r* = 0.3 or strong for *r* = 0.5 (15), and significant with *p* < 0.05.

Additionally, subgroup analysis was performed comparing gender, using a two-sample *t*-test, significant with *p* < 0.05, and different education levels, performing an ANOVA single factor analysis of variance significant with *p* < 0.05.

#### Ethical Approval

The ethical approval has been obtained from the ethical committee of Hamburg Chamber of Physicians (approval number: PV5722).

## Results

### Development and Feasibility Testing

The website's first evaluation was conducted with *n* = 4 MRI experts and *n* = 5 expert patients. The MRI expert group consisted of one neurologist from Italy, Spain, and the UK, respectively, and one neuroradiologist from Germany. Expert patients were women between 35 and 69 years of age. Most interviewees rated subjective MRI knowledge as high. Interviewees perceived the weight put on MRI findings as overestimated in the fields of prognosis and therapy control. Considering the scope of an MRI education for pwMS, expert patients showed ambivalent opinions, ranging from “*not too much information”* to “*as much as possible”*.

The second evaluation round was performed with *n* = 5 pwMS and *n* = 3 expert patients. PwMS were one male and four women aged between 36 and 44 years. MRI subjective knowledge was assessed as medium (*n* = 3) or low (*n* = 2). All pwMS indicated that the content was comprehensive and covered all relevant topics. One patient was only interested in the “Learning to read” section. Most pwMS agreed that UMIMS was a helpful source to get information on MRI in MS; some emphasized that the subject matter was demanding, especially for pwMS with cognitive and visual impairments.

Expert patients were women between 34 and 52 years of age. Interviewees emphasized that the website's content should be voiced in an encouraging tone. They demanded for statements to be put into perspective, e.g., using absolute numbers instead of “often” and to use careful wording, avoiding unproven implications, e.g., “lesion” should not be equated with tissue damage. One comment in this group was that UMIMS lacked information on the importance of MRI in monitoring progressive multifocal leukoencephalopathy risk under immunotherapies.

After each evaluation round, the website was revised accordingly. No chapters were deleted or added to UMIMS. Selected quotes from both rounds of evaluations are shown in Table 1.

**Table 1:** Quotes from audiotaped interviews divided into different (sub-) categories.

**Major category**	**Subcategory**	**Statements**
Primarily evaluation of the website's prototype with MRI experts^a^ *n* = 4 and expert patients^b^ *n* = 5		
*Relevance of MRI in MS*	Diagnosis and outcome measure	**MRI expert**: MRI has been of paramount importance in the field of diagnostic, supporting early diagnosis. It is useful as an outcome measure and to investigate the pathophysiology of the disease.
	Prognostic value	**Expert patient**: I think it is utterly overrated by neurologists, partially also by patients, because there is a very big fear that if it can be seen in the MRI, it immediately means myelin loss, cell death, and that doesn't necessarily have to be true at all.
*MRI training*	Individual approach	**Expert patient**: It depends on how much you can impose on patients with MRI training. It varies greatly. On one hand, it depends on the patient's level of education, but it also majorly depends on which phase of the disease the patient is in.
Evaluation of the advanced website draft with pwMS *n* = 5 and expert patients *n* = 3		
*Overall impression*	Structure of the presentation	**pwMS:** Great are the “by the way” options. [To get additional knowledge input.]
	Practical value	**Expert patient**: (…) quite amazing presenting the sounds of the MRI. (*How does an MRI work)*
	Layout	**Expert patient**: Richer colors and a larger font would be nice. This would be better for people with visual impairments. A function to adjust the size should be placed centrally e.g., in the top corner of the page.
*Module 1:* *Main chapters^c^*	Relevance	**pwMS**: Yes, all topics are relevant for me. *(What can be seen in the MRI)* **pwMS**: The comments on “When should an MRI not be performed?” are not necessary in my opinion. The explanation and the decision are the doctor's responsibility. Regardless of how I interpret my MRI. (*Why MRI)*
	Comprehensibility	**pwMS**: Yes, that was very well explained. *(Why MRI)* **pwMS**: It is already a really complicated topic. It starts with spatial and temporal dissemination. That is already complicated. Until the end, I had difficulties with these terms. But the whole topic is extremely difficult. *(Why MRI)*
	Balanced presentation of information	**pwMS**: Yes, information is very well balanced. *(What does MRI show in MS)*
	MRI images and graphics	**pwMS**: It is very specific, and you need a good eye to see a lesion. Size of the images is ok. *(What can be seen in the MRI)*
*Module 2: Learning to read*	Relevance	**pwMS**: Yes, in any case, it's relevant. You sit in front of it with your own report and want to learn to read it immediately. **Expert patient:** It is very demanding. With a lot of question marks. I don't know how many people would want to use this. My idea: The left side can be written in medical terms just like the doctor phrases it and the right side could contain a translation “for dummies” or in “German”.
	MRI reports	**pwMS**: Yes, I think that's a good thing. I do not miss anything. **pwMS**: Very difficult. (...) Either text or video next to it with a person explaining the content. I believe something visual with a version for 3-year-olds would be very helpful. The final points are very technical.
	Example MRI images	**pwMS**: The selection of MRI images is adequate. **pwMS**: I am most interested in understanding my report and my images and as a result this platform provides me with enough information that I am able to do that.
	MRI evaluation schemes	**pwMS**: Basically, everything is understandable, nevertheless one stands there rather lost. But it is also very interesting. Under guidance, it's certainly even more fun.
*Module 3: Training*	General appraisal	**pwMS**: I liked it quite a lot. I left out the expert quizzes, which were too much of a chore for me. **pwMS**: In principle, all questions are clearly solvable for us. However, subject-specific questions already cause problems since technical terms/knowledge are not available or have not yet been consolidated.

*pwMS, people with MS*.

a *Physicians (neurologists, radiologists)*.

b *People affected by MS, who include experiential (i.e., personal and collective) knowledge of the illness as well as pertinent awareness of diseases and academic involvement*.

c *If the quote refers to a specific chapter, the chapter is given in parentheses behind*.

### Pilot Study

In the pilot study, *n* = 261 pwMS fulfilled eligibility criteria, answered the baseline questionnaire, and received access to UMIMS. The follow-up questionnaire was answered by *n* = 122 participants; after exclusion of participants, who did not provide demographic data, *n* = 92 pwMS remained (finisher 35.2%) (see Table 2).

**Table 2:** Demography of the pilot cohort.

	**Pilot cohort *n* = 92^a^**
Women (%)	71.7
Age in years	42.2 (10.5)
Disease course (%)	
Primary manifestation (%)	4.3
RRMS (%)	72.8
SPMS (%)	12.0
PPMS (%)	4.3
Unclear	6.5
Time since diagnosis in years	6 (4.5)
Mean level of disability (PDDS)	2 (2)
Education (%)	
Highschool degree	66.3
Secondary degree	29.3
No degree/primary degree	4.3
Number of received MRIs	
<5	28.3
5 to 10	40.2
>10	31.5

*RRMS, relapsing-remitting multiple sclerosis; SPMS, secondary-progressive multiple sclerosis; PPMS, primary-progressive multiple sclerosis; PDDS, Patient Determined Disease Steps*.

a *Mean value (standard deviation)*.

#### Acceptance

**Figure 2:**
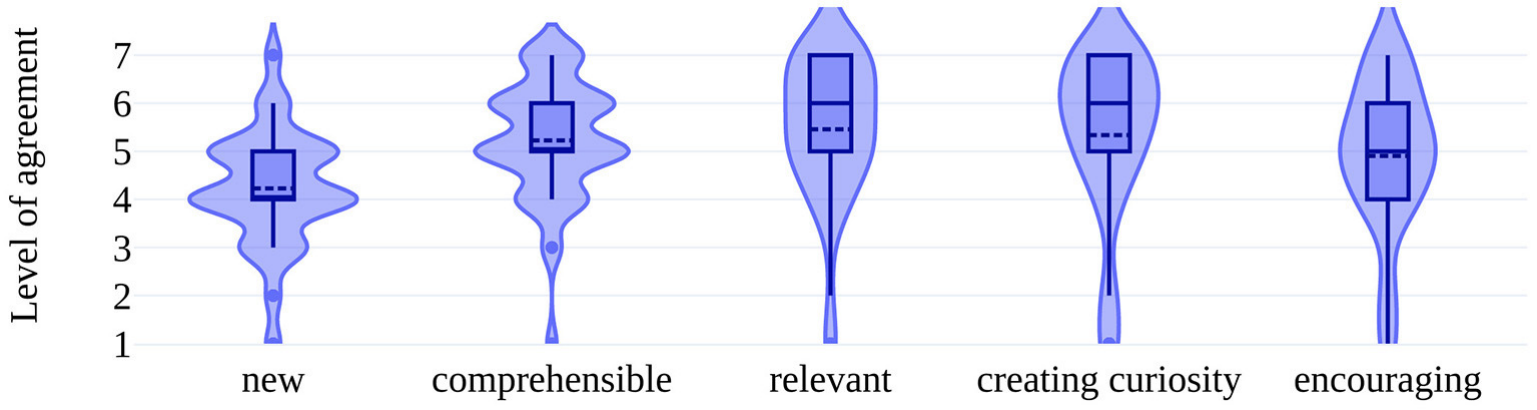
Participants' website experiences. Data in violin plots. The level of agreement on the *y*-axis was assessed on a 7-point Likert-scale ranging from 1 = very low agreement to 7 = very high agreement. Categories are shown on the *x*-axis. The extension from the center line is proportional to the density of data to the given *y*-value. The box plot inside gives upper and lower adjacent values, interquartile ranges, median and shows outliners (light blue dots). Additionally, the mean is shown with a dark blue dotted line.

Participants rated the information on UMIMS as comprehensible (mean 5.2/7, *SD* 1.2), creating curiosity (mean 5.3/7, *SD* 1.7), and relevant (mean 5.5/7, *SD* 1.5). Comprehensibility correlated with MRI risk-knowledge, i.e., MRI-RIKNO 2.0 scores (Pearson coefficient 0.36, *p* < 0.05). For most participants, the information was new (mean 4.2/7, *SD* 1.2). The content encouraged rather than unsettled participants (mean 4.9, *SD* 1.6) (see Figure 2).

**Figure 3:**
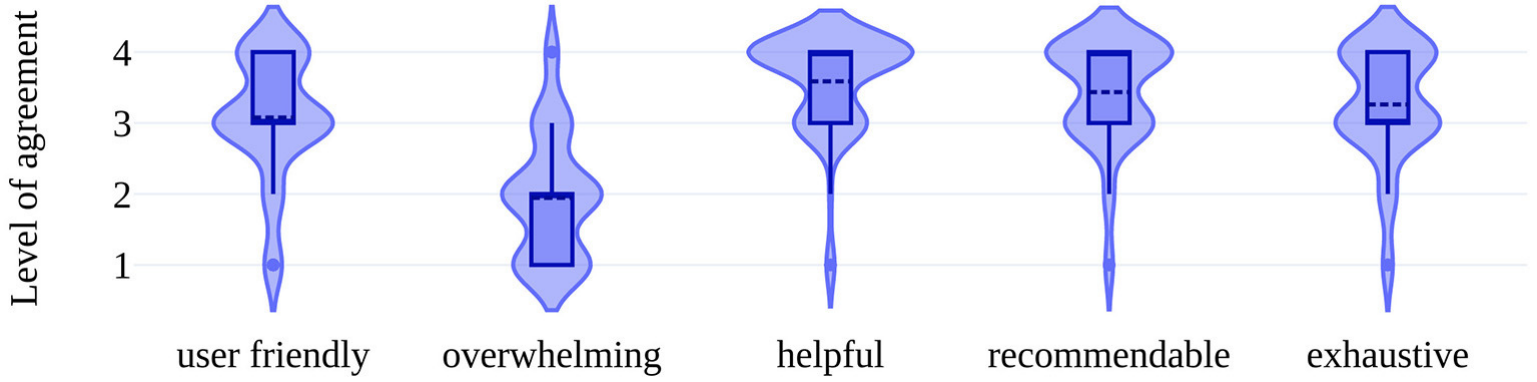
Participants' perception of the website. Violin plots show the distribution of answers (for violin plot detail see Figure 2). This figure shows the level of agreement on a 4-point Likert-scale ranging from 1 = low agreement to 4 = high agreement to the categories seen on the *x*-axis. Note the 4-point format to push participants to take positions and avoid neutral answers.

Participants assessed the website as user-friendly (mean 3.1/4, *SD* 0.8). The content was rated as helpful in the understanding of MRI in MS (mean 3.6/4, *SD* 0.7) and most would recommend UMIMS to other pwMS and relatives (mean 3.4/4, *SD* 0.7). For most participants, UMIMS contained all relevant information on MRI in MS (mean 3.3, *SD* 0.8), without being too extensive (mean 2.0/4, *SD* 0.9) (see Figure 3).

Most participants spent 1–4 h on the website (53.3%); 20.7% spent <1 h and 20.6% more than 4 h. A small percentage did not look at the website at all (5.4%). Time spent on the website correlated with the MRI risk-knowledge (Pearson-coefficient = 0.33, *p* < 0.05).

#### Evaluation of Voluntary Feedback

Participants left 49 voluntary comments: 31 contained positive appraisals, 22 were neutral and 13 were critical comments. Appraisals were mainly the expressions of gratitude. Neutral comments were mostly related to technical issues, e.g., broken hyperlinks. Criticism was directed at the color spectrum used, at for some to scientific contents and the structure of the website as some participants had difficulties to navigate, e.g., it was occasionally noted that after visiting the website again, it was not always easy to find previously read information due to the abundance of information.

In the following, the website was revised again, e.g., navigation was made easier by showing users which chapter and lesson they are currently working in.

#### MRI-Risk Knowledge

On an average, participants of the pilot cohort, i.e., with access to UMIMS, achieved 72.0% correct answers in the MRI-RIKNO 2.0. In comparison, a study population from an online survey of 2016 with n = 508 pwMS *without* MRI education scored an average of 62.1% correct answers, i.e., 9.9% points lower. In 20 out of 22 questions, the pilot cohort achieved higher scores. The biggest differences were found in one question about the contrast medium frequently used in MRI for pwMS (+ 22.5% in the pilot cohort) and one in which participants had to assign periventricular lesions on an MRI image (+ 23% in the pilot cohort).

In a subgroup analysis for gender, no difference on MRI-RIKNO 2.0 scores between women and men could be detected.

Patients with “no degree/ primary degree” showed 64.8% correct replies, patients with “secondary degree” education level 68.7%, and pwMS with “high school degree” had 74% correctly answered questions. However, despite the descriptive difference ANOVA analysis of variance showed no significant difference between the groups (*p* > 0.05).

#### Subjective Knowledge

Subjective MRI knowledge improved significantly during the study: Before using UMIMS, participants rated their knowledge at 3.9/7 (*SD* 1.5), afterward at 4.3/7 (*SD* 1.2, *p* < 0.05). A moderate correlation was found between post-intervention subjective knowledge and objective knowledge, i.e., MRI-RIKNO 2.0 scores (Pearson-coefficient = 0.4, *p* < 0.05).

In a subgroup analysis, male pwMS estimated their subjective MRI knowledge before using UMIMS with a mean of 4.2/7 (*SD* 1.5) significantly better than female pwMS with a mean of 3.7/7 (*SD* 1.4) (*p* < 0.05). In the assessment of post-intervention subjective knowledge, no significant difference between genders was found matching the MRI-RKNO 2.0 scores.

#### Emotions and Attitude Toward MRI

For the MRI-EMA subscale “Fear of MRI scan” a mean of 1.82 (*SD* 0.9) out of 4 was scored, thus the MRI examination itself did not cause relevant anxiety; MRI results tended to cause more anxiety (subscale “Fear of MRI results,” mean 2.2, *SD* 0.8). Receiving MRI results provided pwMS with a feeling of control over their disease (subscale “Feeling of control,” mean 2.6, *SD* 0.8) and participants generally felt competent to discuss their MRI with their physician (subscale “Feeling of competence,” mean 2.8, *SD* 0.8). When asked directly, whether participants felt competent to discuss their MRI with a physician, 66.3% agreed (Likert-scores 3 and 4) compared to only 46% in the online cohort (10).

In a subgroup analysis for gender significant differences were found. For the subscale “Fear of MRI scan” male pwMS scored an average of 1.5/4 (*SD* 0.7), female pwMS scored an average of 2/4 (*SD* 0.9) (*p* < 0.05). For the subscale “Feeling of control” male pwMS scored an average of 3/4 (*SD* 0.7) female pwMS scored an average of 2.7/4 (*SD* 0.8) (*p* < 0.05). Male pwMS tended to be more confident in their knowledge, whereas female pwMS tended to be more anxious about the MRI procedure.

## Discussion

Recognizing the paucity of patient-information programs about MRI, this study addressed the development of an educational website on all aspects of MRI in MS. Qualitative evaluation indicated high usability. The possibility of some basic training to read MRI and to understand MRI reports was highly appreciated. Quantitative results of the pilot survey indicated good acceptance: UMIMS received great appraisal from users, who rated it as user-friendly, exhaustive, but not overwhelming content-wise, helpful, and recommendable for other pwMS. As another strong indicator of acceptance half of the patients spent 1 to 4 and 21% more than 4 h on the website. Subsequently, high satisfaction of the participants led to minor adjustments of the website.

In an MRI-risk knowledge questionnaire administered after using the website, 72% of questions were answered correctly. Thus, compared to a previous cohort without MRI education, with 62% of questions answered correctly, participants scored higher (8). Concordantly, subjective MRI knowledge improved significantly after using UMIMS. Considering that objective and subjective MRI knowledge showed a modest correlation in the pilot cohort, we hypothesize that using UMIMS has the potential to significantly improve MRI risk-knowledge of pwMS. However, another study points out, knowledge alone is not sufficient for patients to participate in shared decision making, they also need “power,” i.e., confidence (16). UMIMS delivered just this. While only 46% of pwMS without MRI education felt competent to discuss MRI findings with their physicians, 66.3% of participants of the UMIMS pilot cohort did so (10).

Previously, pwMS stated, that their MRI results provided them with a feeling of control over their disease, (10) i.e., they perceived their MRI as a “mirror of their disease” –a sentiment that, taking the current state of scientific knowledge into account, is unjustified (17). Good MRI knowledge, i.e., a better understanding of the limitations of MRI, was linked to a lower feeling of control (8). However, even though in this study, pwMS were extensively educated about MRI, they still stated, that their MRI results provided them with a feeling of control over their disease. The adherence to this flawed belief might be a reflection of their real-world experiences in which neurologists often put immense emphasis on MRI results as demonstrated in another study: In an analysis of 8,311 MRIs in pwMS from an aggregated registry, disease-modifying therapy (DMT) was initiated or changed in 27% of cases following the appearance of even only one silent lesion (i.e., isolated radiological activity without signs of a clinical relapse) (18). However, premature immunotherapy change may lead to an unnecessarily rapid therapy escalation, with pwMS facing serious side effects of DMTs reserved for aggressive disease courses. This, again, highlights the importance of a critical appraisal of MRI results–by patients and physicians.

Little research has been done on patient education about radiological investigations. Bowden et al. (19) did study the quality of general radiology-related healthcare information on the internet and found very poor quality. Some work exists on the improvement of patient compliance for radiologic procedure using informative videos (20). Similar findings were recently reported by Yakar et al. (21) and Bolejko et al. (22) indicating that written or video information reduced state anxiety. Hyde et al. (23) asked patients from general radiology undergoing CT or MRI scanning for unmet needs in relation to the conduct of these procedures, summarizing that overall patients indicated having received too little information. All these studies focused on the scanning procedure to decrease anxiety and increase the efficacy of image acquisition in general radiology–to our knowledge, no study has addressed pwMS' understanding and weighting of image findings. In areas with easy access, MRI screening investigations are nowadays performed extensively. This inevitably leads to incidental findings and considerable diagnostic and therapeutic dilemmas such as the radiologically isolated syndrome (RIS) in the MS context (24). Therefore, a deepened understanding among pwMS of why and when to perform imaging studies, as well as possible consequences, is highly warranted. However, the attitudes of treating neurologists to discuss with patients issues raised by a higher degree of knowledge are highly relevant as they could be a potential barrier for the implementation of more autonomous role preferences of pwMS. Neurologists' attitudes are briefly captured in the currently running RCT on UMIMS and need to be focused on more in future studies.

In this study, subgroup analysis revealed some significant differences in outcomes between women and men. Male pwMS tended to be more confident in their knowledge as the feeling of control over the disease and subjective MRI knowledge before using UMIMS was perceived higher in this group. Female pwMS were more anxious about the MRI examination itself. These findings suggest that there might be gender-specific differences and needs in online education that might be analyzed with more attention in future studies.

In addition to gender differences, UMIMS should still be improved and adapted to address intercultural differences, e.g., by changes in design, scope of videos, and autodidactic content, and be more inclusive toward pwMS with severe health impairments and illiterate patients.

Limitations of the study were the low completion rate (35.2%) and the online recruitment process which might have led to selection bias toward those who were more comfortable using online resources, and toward those who are more inclined to proactively seek out information or educational opportunities. Additionally, the mean level of disability (2 on the PDDS) was low, which might be partially explained by the online recruitment process as well, i.e., pwMS must be familiar with internet use and tend to be younger patients, but probably also by the fact that the need for information is particularly high in earlier stages of the disease.

Overall, this study showed that the education website UMIMS meets pwMS' information needs on the complex topic of MRI in MS management. It has the potential to increase pwMS' MRI risk-knowledge, and thereby possibly enhance participation in MRI-based medical decisions. To verify this hypothesis, a multicentred randomized, controlled, double-blind trial is currently running (NCT03872583).

## Data Availability Statement

The raw data supporting the conclusions of this article will be made available by the authors, without undue reservation.

## Ethics Statement

The studies involving human participants were reviewed and approved by the Ethical Committee of Hamburg Chamber of Physicians (approval number: PV5722). The patients/participants provided their written informed consent online to participate in this study.

## Author Contributions

MF and IS conducted the interviews during the website's development process, periodically revised the website, managed the data collection, and analyzed qualitative interview data as well as quantitative data of the pilot study. They jointly prepared the draft of the paper. AR participated in regular evaluations of the development and piloting process of UMIMS, provided crucial input, particularly on methodological implementation and data evaluation as well as for the manuscript. DC and CL were actively involved in the development of the website as an MRI expert as well as in proofreading the paper. JS was actively involved in the development of the website as an expert patient as well as in proofreading the paper. AS participated in regular evaluations of the development and piloting process of UMIMS, provided crucial input, particularly on methodological implementation and data evaluation. CH developed the initial concept of the intervention, conducted the MRI expert interviews and is head of the working group in which this study was planned, developed and evaluated. He revised parts of the manuscript. All authors contributed to the article and approved the submitted version.

## Funding

This project was funded by Sanofi Genzyme. The authors declare that this study received funding from Sanofi Genzyme. The funder was not involved in the study design, collection, analysis, interpretation of data, the writing of this article or the decision to submit it for publication.

## Conflict of Interest

IS has received funding for academic conferences by Sanofi Genzyme. AS has received funding from Roche Pharma. CL received a research grant by the German Federal Ministry for Education and Research, BMBF, German Competence Network Multiple Sclerosis (KKNMS), grant no. 01GI1601I, has received consulting and speaker's honoraria from Biogen Idec, Bayer Schering, Daiichi Sanykyo, Merck Serono, Novartis, Sanofi, Genzyme, and TEVA. DC is a consultant for Biogen and Hoffmann-La Roche. He has received research funding from Hoffmann-La Roche, the International Progressive MS Alliance, the MS Society, and the National Institute for Health Research (NIHR) University College London Hospitals (UCLH) Biomedical Research Centre. He co-supervises a clinical fellowship at the National Hospital for Neurology and Neurosurgery, London, which is supported by Merck. CH received research grants and speaker's honoraria from Biogen, Bristol Myers Squibbs, Merck, Novartis, Roche. The remaining authors declare that the research was conducted in the absence of any commercial or financial relationships that could be construed as a potential conflict of interest.

## Publisher's Note

All claims expressed in this article are solely those of the authors and do not necessarily represent those of their affiliated organizations, or those of the publisher, the editors and the reviewers. Any product that may be evaluated in this article, or claim that may be made by its manufacturer, is not guaranteed or endorsed by the publisher.
