# Supplementary material for: Understanding Magnetic Resonance Imaging in Multiple Sclerosis (UMIMS): Development and Piloting of an Online Education Program About Magnetic Resonance Imaging for People With Multiple Sclerosis
Source: Front Neurol. 2022 Mar 28;13:856240. doi: 10.3389/fneur.2022.856240 (PMC8996193; doi:10.3389/fneur.2022.856240)
Supplement: Supplementary file 1 [file Table_1.docx]

**Supplementary material**

| **Table 1.** Development of the chapters of UMIMS. | | | |
| --- | --- | --- | --- |
|  | first version | second version | final version |
| About MRI ^a^ |  |  |  |
| The website ^b^ |  |  | ^d^ |
| structure ^c^ |  |  |  |
| special functions |  |  |  |
| What is MS? |  |  |  |
| Why MRI? |  |  |  |
| diagnosis |  |  |  |
| prognosis |  |  |  |
| therapy control |  |  |  |
| bouts |  |  |  |
| How does an MRI work? |  |  |  |
| procedure |  |  |  |
| contrast medium |  |  |  |
| What can be seen in the MRI? |  |  |  |
| brain anatomy |  |  |  |
| networks |  |  |  |
| typical MS symptoms |  |  |  |
| MRI sequences |  |  |  |
| What does the MRI show for MS? |  |  |  |
| lesions in the MRI |  |  |  |
| atrophy and black holes |  |  |  |
| Extra knowledge |  |  |  |
| physics of the MRI scanner |  |  |  |
| What is a diagnostic test? |  |  |  |
| new technologies |  |  |  |
| Learning to read |  |  |  |
| Part 1: MRI-report |  |  |  |
| Part 2: MRI-results |  |  |  |
| Part 3: Read your MRI |  |  |  |
| Abbreviations: UMIMS, Understanding magnetic resonance imaging in multiple sclerosis.  ^a^ Section.  ^b^ Main chapter.  ^c^ Subchapter.  ^d^ Light grey colour indicates the adding of the (sub-)chapter. | | | |
